# Decoding induced mesenchymal stem cells functionality: why differentiation protocols matter

**DOI:** 10.3389/fcell.2026.1792744

**Published:** 2026-02-24

**Authors:** Prakash Gangadaran, Ramya Lakshmi Rajendran, Sathish Muthu, Byeong-Cheol Ahn

**Affiliations:** 1 Department of Nuclear Medicine, School of Medicine, Kyungpook National University, Daegu, Republic of Korea; 2 Cardiovascular Research Institute, Kyungpook National University, Daegu, Republic of Korea; 3 BK21 FOUR KNU Convergence Educational Program of Biomedical Sciences for Creative Future Talents, Department of Biomedical Sciences, School of Medicine, Kyungpook National University, Daegu, Republic of Korea; 4 Central Research Laboratory, Meenakshi Medical College Hospital and Research Institute, Meenakshi Academy of Higher Education and Research, Kanchipuram, Tamil Nadu, India; 5 Department of Orthopaedics, Orthopaedic Research Group, Coimbatore, Tamil Nadu, India; 6 Department of Nuclear Medicine, Kyungpook National University Hospital, Daegu, Republic of Korea

**Keywords:** differentiation protocols, induced mesenchymal stem cells, mitochondrial profiling, regenerative medicine, standardization

## Outline

The recent article by Ababneh et al. (2025) provides a timely and valuable contribution to the evolving discussion on protocol-dependent variability in induced mesenchymal stem cells (iMSCs). By systematically comparing five distinct differentiation strategies (Table 1), the authors highlight a crucial yet underexplored aspect of stem cell biology: the operational definition of functional MSCs and the insufficiency of surface markers alone to predict cellular behavior. This opinion builds upon the findings of Ababneh et al. (2025) and advocates for a paradigm shift in the definition, standardization, and manufacture of iMSCs for regenerative medicine applications.

**TABLE 1:** Five iMSC generation methods.

iMSCs	Methods
iMSC1	iPSCs cultured on Matrigel, then switched to α-MEM with 10% FBS and 10 µM SB431542 for 14 days, followed by expansion in α-MEM without SB431542
iMSC2	iPSCs were directly switched to α-MEM containing 10% FBS, 1% GlutaMAX, nonessential amino acids, and 2% penicillin/streptomycin
iMSC3	iPSCs were maintained in mTeSR1 with 10 μM SB431542, with daily medium change for 4–5 weeks until MSC-like morphology appeared
iMSC4	iPSCs sequentially treated with activin A, CHIR99021, FGF2, BMP4, Y27632, follistatin, PDGF, EGF, and ascorbic acid for mesoderm to MSC differentiation
EB-iMSC	iPSCs formed embryoid bodies in α-MEM with 15% FBS with retinoic acid on days 2 and 4, then cultured on Matrigel with bFGF from day 12

Abbreviations: α-MEM, minimum essential medium alpha modification; FBS, fetal bovine serum; GlutaMAX, L-alanyl-L-glutamine supplement; SB431542, TGF-β, inhibitor; FGF2, Fibroblast growth factor 2; BMP4, Bone morphogenetic protein 4; PDGF, Platelet-derived growth factor; EGF, epidermal growth factor; bFGF, basic fibroblast growth factor; EB, embryoid body; iPSC, induced pluripotent stem cell; iMSC, induced mesenchymal stem cell.

### Protocols define cell identity beyond markers

Ababneh et al. (2025) demonstrate that all five iMSC protocols (Table 1) yield cells that satisfy the 2006 International Society for Cellular Therapy (ISCT) minimal criteria, including adherence to plastic, expression of cluster of differentiation (CD) markers CD90, CD105, CD73, CD44, and trilineage differentiation potential. Although the protocols demonstrated phenotypic consistency, they differed markedly in functional properties, highlighting intrinsic functional heterogeneity. Despite expressing canonical MSC surface markers, each iMSC type exhibits distinct differentiation, proliferation, colony formation, mitochondrial function, redox balance, senescence, and migratory profiles. Notably, iMSC4 displayed attenuated CD105 expression yet retained strong osteogenic and proliferative potential, challenging the assumption that surface antigens reliably predict potency. Conversely, iMSC2 and embryoid body-derived (EB)-iMSC, although marker-positive, exhibited pronounced osteogenic dominance but adipogenic deficiency. A consistent osteogenic bias was observed across all iMSC lines, particularly pronounced in EB-iMSC and iMSC2, accompanied by suppressed expression of adipogenic genes (proliferator-activated receptor gamma [PPARG] and ADIPSIN). Retinoic acid, used in EB-iMSC induction, is known to inhibit adipogenesis via PPARγ repression, steering cells toward osteogenesis, whereas the omission of laminin-511 substrates in iMSC2 may deprive cells of matrix cues critical for adipogenic specification (Kim and Ko, 2014; Schwarz et al., 1997; Hisada et al., 2013). These directed differentiation biases are not artifacts; rather, they could be strategically exploited, for example, using osteo-favored iMSCs for bone repair or adipogenesis-competent iMSCs for metabolic or reconstructive applications. Functionally, iMSC4 exhibited the greatest proliferative capacity, reflecting enhanced metabolic activity and viability. In contrast, iMSC3 displayed superior clonogenicity, suggesting stronger self-renewal potential despite a lower proliferation rate. Notably, mitochondrial performance and reactive oxygen species (ROS) homeostasis correlated with iMSC quality. Mitochondrial membrane potential (MMP) analysis revealed compromised bioenergetics in iMSC3 and iMSC4, yet these cells demonstrated resistance to carbonyl cyanide m-chlorophenyl hydrazone (CCCP)-induced depolarization, indicating adaptive metabolic remodeling rather than dysfunction. Such resilience may reflect enhanced mitophagy or altered glycolytic flux, phenomena increasingly recognized as hallmarks of stem cell robustness (Lin et al., 2021). All iMSC types exhibited significantly lower ROS accumulation than bone marrow MSCs following oxidative challenge, consistent with intrinsic antioxidant defense. Furthermore, each iMSC type exhibited distinct senescence and migratory behaviors. Higher levels of senescence were observed in iMSC1 and iMSC4 compared with iMSC2, iMSC3, and iMSC5. In contrast, iMSC3 and iMSC4 displayed reduced migration at 48 h. These findings underscore that antigenic profiles alone are insufficient surrogates for cellular potency, consistent with previous studies that emphasize the primacy of epigenetic and metabolic reprogramming over phenotypic confirmation in defining iMSC functional identity (Wruck et al., 2021; Xu et al., 2019).

The 2025 Delphi-driven consensus for MSC characterization explicitly discourages reliance on plastic adherence and trilineage differentiation as defining criteria, recommending instead functional potency assays and tissue-of-origin specification (Renesme et al., 2025). Ababneh et al. (2025) findings provide empirical justification for this update: although all iMSCs were ISCT-compliant, they diverged in mitochondrial health, redox resilience, senescence, and motility—attributes directly linked to therapeutic efficacy. These results necessitate a critical reassessment of the long-standing notion that MSC identity can be adequately defined by surface markers alone. Differentiation protocols do not merely produce cells that “fit” MSC checklists; they also confer lineage memory and functional fate.

Beyond protocol-dependent variability, the genetic background of the parental iPSCs represents a critical determinant of iMSC functionality. Recent evidence demonstrates that iMSCs derived from different somatic origins exhibit distinct genetic and epigenetic signatures, which in turn influence lineage bias, senescence, and immunomodulatory properties (Wang J. et al., 2024). This underscores that functional heterogeneity is not solely a product of differentiation methodology but also reflects donor cell identity and reprogramming history. Future frameworks for iMSC standardization must therefore integrate both protocol-specific and source-specific variables to ensure reproducibility and therapeutic reliability.

### Clinical relevance and translational implications

From a clinical perspective, the findings by Ababneh et al. (2025) have direct implications for regenerative medicine, particularly in orthopedics, cardiovascular repair, and immune modulation. The observed protocol-dependent variability in osteogenic and adipogenic differentiation is not merely theoretical; it dictates therapeutic suitability. For instance, EB-iMSCs and iMSC2, with osteogenic bias and suppressed adipogenesis, may be ideal candidates for bone regeneration in osteoporotic fractures or spinal fusion, where adipogenic drift is undesirable (Chiou et al., 2023). Conversely, protocols that preserve adipogenic potential may be more appropriate for soft tissue engineering or metabolic disease modeling (Chiou et al., 2023).

Reduced senescence and enhanced ROS resistance across most iMSC lines suggest a rejuvenated cellular phenotype, potentially improving engraftment, survival, and paracrine efficacy *in vivo*. However, the diminished migratory capacity observed in iMSC3 and iMSC4 raises concerns for applications requiring homing to injury sites, such as myocardial infarction or inflammatory arthropathies. These functional nuances highlight the necessity of aligning protocol selection with specific clinical endpoints (Wu et al., 2024).

In the context of Good Manufacturing Practice (GMP) and clinical-grade cell production, the study underscores the need for potency assays that reflect intended therapeutic mechanisms, whether immunomodulation, angiogenesis, or matrix remodeling (Galipeau et al., 2016). Without such alignment, phenotypically compliant but functionally suboptimal iMSCs may be deployed in clinical trials, potentially compromising efficacy and reproducibility. In addition to cellular applications, exosomes secreted during iMSC induction and expansion represent a promising therapeutic adjunct. These extracellular vesicles carry bioactive cargo, including proteins, lipids, and RNAs, that can modulate immune responses, promote angiogenesis, and enhance tissue repair.

Wang J. et al. (2024) highlighted that iMSC-derived exosomes may provide a cell-free alternative with reduced safety concerns related to engraftment or tumorigenicity. Incorporating exosome profiling into potency assays and omics-guided quality control could expand the translational utility of iMSCs and strengthen their role in regenerative medicine. Finally, the transition toward animal-free culture systems is essential for clinical translation. Reliance on fetal bovine serum (FBS) and other xenogeneic supplements introduces variability and regulatory hurdles. The development of chemically defined, xeno-free, and GMP-compatible media formulations will be critical to ensure reproducibility, safety, and compliance with international standards. Incorporating animal-free expansion strategies into iMSC manufacturing pipelines will accelerate their progression from experimental models to clinical-grade therapeutics.

Although exosome-based therapies are considered theoretically safer and more scalable than cell-based products, they can exhibit significant biological heterogeneity. The composition of exosomes varies depending on the parental cell state, passage number, culture conditions, and manufacturing parameters, all of which can significantly influence their cargo and bioactivity. Additionally, regulatory classification under Advanced Therapy Medicinal Product (ATMP) frameworks remains unclear, and standardized potency assays for exosomes are still lacking—posing a major translational challenge (Tan et al., 2024; Wang C-K. et al., 2024).

### Ethical and regulatory considerations

Ethical sourcing and donor traceability are critical for iPSC-derived iMSC manufacturing. Since iMSCs inherit epigenetic memory from parental iPSCs, donor consent frameworks must explicitly address genomic editing implications, long-term data use, and cross-border sourcing. Donor screening for infectious and genetic risks is essential to ensure therapeutic reliability. Regulatory oversight should mandate transparent documentation of donor identity, consent, and traceability to safeguard both patient safety and public trust.

### Precision in iMSC manufacturing

Ababneh et al. (2025) comparative framework enables rational protocol selection; however, variability persists across donor iPSC sources, passage number, matrix composition, and small-molecule cues. The field urgently requires GMP-compatible differentiation roadmaps integrating phenotypic profiling with multi-omic quality control. Single-cell transcriptomics and proteomics can reveal subpopulation heterogeneity and markers of potency decline or lineage drift. Incorporating these insights into standardized workflows could transition iMSC production from artisanal to reproducible bioprocesses. Furthermore, functional equivalence testing against clinically validated MSCs should be mandatory to ensure therapeutic reliability (Trounson and McDonald, 2015).

Protocol harmonization alone cannot resolve translational hurdles. Large-scale bioreactor expansion introduces metabolic shifts and lineage memory persistence that may compromise potency. Cost-effectiveness compared to primary MSCs remains uncertain, and long-term safety monitoring is indispensable to detect late-onset risks. Addressing these bottlenecks requires integrated bioprocess engineering, real-time metabolic monitoring, and rigorous post-transplant surveillance.

### Unresolved issues

While Ababneh et al. (2025) meticulously control for culture conditions, certain questions remain unresolved.

- Limited diversity and assay bias: The reliance on only two donor iPSC lines and scratch-based migration assays limits the biological and functional interpretation of the findings.
- Translational uncertainty: The study presents only *in vitro* results, such as low ROS accumulation and reduced senescence. These observations require *in vivo* validation to confirm cellular stability, differentiation capacity, and regenerative relevance.
- Definitional transition: The study bridges the 2006 ISCT MSC criteria and the 2025 updated guidelines, highlighting the ongoing shift toward function-based evaluation.

## Conclusions and future direction

Ababneh et al. (2025) provide a foundational comparative atlas of iMSC differentiation pathways, demonstrating that differentiation protocols, rather than markers, dictate function. Their findings support a critical paradigm shift in stem cell science: cell identity is dynamic, context-dependent, and encoded by differentiation protocols. This study underscores that the development of clinical-grade iMSCs should prioritize metabolic and functional characterization over the expression of phenotypic markers. By adopting this framework, the field can advance beyond descriptive phenotyping toward reproducible, precision-engineered stem cell therapeutics.

Emerging platforms such as organoid systems, microfluidic organ-on-a-chip models, and AI (artificial intelligence)-assisted multiparametric analytics may further enhance the predictive assessment of iMSC functionality and protocol refinement. Given the technical and computational complexity of these approaches, their systematic evaluation warrants dedicated discussion in future focused reviews.
